# Lipid Droplets in Endosymbiotic *Symbiodiniaceae* spp. Associated with Corals

**DOI:** 10.3390/plants13070949

**Published:** 2024-03-25

**Authors:** Buntora Pasaribu, Noir Primadona Purba, Lantun Paradhita Dewanti, Daniel Pasaribu, Alexander Muhammad Akbar Khan, Syawaludin Alisyahbana Harahap, Mega Laksmini Syamsuddin, Yudi Nurul Ihsan, Sofyan Husein Siregar, Ibnu Faizal, Titin Herawati, Mohammad Irfan, Timbul Partogi Haposan Simorangkir, Tonni Agustiono Kurniawan

**Affiliations:** 1 Department of Marine Science, Faculty of Fisheries and Marine Science, Universitas Padjadjaran, Bandung 40600, Indonesia; noir.purba@unpad.ac.id (N.P.P.); syawaludin.alisyahbana@unpad.ac.id (S.A.H.); mega.syamsuddin@unpad.ac.id (M.L.S.); yudi.ihsan@unpad.ac.id (Y.N.I.); ibnu.faizal@unpad.ac.id (I.F.); 2 Shallow Coastal and Aquatic Research Forensic (SCARF) Laboratory, Faculty of Fishery and Marine Science, Universitas Padjadjaran, Bandung 40600, Indonesia; 3 Department of Plant Biology and Pathology, Rutgers, The State University of New Jersey, New Brunswick, NJ 08901, USA; titin.herawati@unpad.ac.id; 4 Department of Fisheries, Faculty of Fisheries and Marine Science, Universitas Padjadjaran, Bandung 40600, Indonesia; lantun.paradhita@unpad.ac.id; 5 Faculty of Law, Social, and Political Sciences, Universitas Terbuka, Tangerang 15437, Indonesia; daniel.pasaribu@ecampus.ut.ac.id; 6 Tropical Marine Fisheries Undergraduate Programme for Pangandaran Campus, Faculty of Fisheries and Marine Science, Universitas Padjadjaran, Bandung 40600, Indonesia; alexander.khan@unpad.ac.id; 7 Department of Marine Science, Faculty of Fisheries and Marine Science, Universitas Riau, Pekanbaru 28291, Indonesia; sofyan.siregar@lecturer.unri.ac.id; 8 Master Program of Marine Conservation, Faculty of Fisheries and Marine Science, Universitas Padjadjaran, Bandung 40600, Indonesia; 9 Plant Biology Section, School of Integrative Plant Science, Cornell University, Ithaca, NY 14850, USA; mi239@cornell.edu; 10 Faculty of Military Pharmacy, Indonesia Defense University, Bogor 16810, Indonesia; tphsimorangkirsimon4@gmail.com; 11 Collage of Environment and Ecology, Xiamen University, Xiamen 361102, China; tonni@xmu.edu.cn

**Keywords:** lipid droplet, coral, proteins, Symbiodiniaceae species, storage energy

## Abstract

Symbiodiniaceae species is a dinoflagellate that plays a crucial role in maintaining the symbiotic mutualism of reef-building corals in the ocean. Reef-building corals, as hosts, provide the nutrition and habitat to endosymbiotic Symbiodiniaceae species and Symbiodiniaceae species transfer the fixed carbon to the corals for growth. Environmental stress is one of the factors impacting the physiology and metabolism of the corals-dinoflagellate association. The environmental stress triggers the metabolic changes in Symbiodiniaceae species resulting in an increase in the production of survival organelles related to storage components such as lipid droplets (LD). LDs are found as unique organelles, mainly composed of triacylglycerols surrounded by phospholipids embedded with some proteins. To date, it has been reported that investigation of lipid droplets significantly present in animals and plants led to the understanding that lipid droplets play a key role in lipid storage and transport. The major challenge of investigating endosymbiotic Symbiodiniaceae species lies in overcoming the strategies in isolating lesser lipid droplets present in its intercellular cells. Here, we review the most recent highlights of LD research in endosymbiotic Symbiodiniaceae species particularly focusing on LD biogenesis, mechanism, and major lipid droplet proteins. Moreover, to comprehend potential novel ways of energy storage in the symbiotic interaction between endosymbiotic Symbiodiniaceae species and its host, we also emphasize recent emerging environmental factors such as temperature, ocean acidification, and nutrient impacting the accumulation of lipid droplets in endosymbiotic Symbiodiniaceae species.

## 1. Introduction

Symbiodiniaceae species is known as a dinoflagellate organism that resides inside other organisms (hosts) and establishes mutualistic symbiosis [1,2]. Symbiodiniaceae species are divided into nine clades (A-I), with corals associating with different Symbiodiniaceae species depending on environmental conditions and spatial distribution [2]. The association of Symbiodiniaceae species has been observed in various invertebrates such as corals, sea anemones, jellyfish, foraminifera, zoanthids, sponges, and giant clams [3]. The stable mutualistic relationship between corals and Symbiodiniaceae species depends on metabolic and nutritional interactions for coral health in marine ecosystems [4]. Endosymbiotic Symbiodiniaceae species reside within intracellular cells and contribute to releasing inorganic compounds of photosynthesis products as fixed organic carbon to support coral metabolism and growth. In return, the host provides nutrient supply, protection, and a stable environment to Symbiodiniaceae species [3]. The mutual exchange of photosynthesis products by Symbiodiniaceae species to the hosts and regulation of necessary nutrient occurrence during the association demonstrate the survival of coral reefs in tropical seas [5,6]. However, the disruption of the symbiotic relationship caused by the expulsion of symbionts into the tissue layers of coral and the excessive loss of symbionts associated with the degradation of photosynthesis pigment, known as the bleaching process, becomes a driver of coral mortality [7]. It has been elucidated that most of the Symbiodiniaceae species are found within the symbiosome membrane in the gastrodermal cell layer. The most common form of Symbiodiniaceae species is described as a coccoid cell, brown in color, with a diameter of 5 to 15 µm. However, the characteristics of Symbiodiniaceae species can be affected by culture phase, nutrient exposure in hospite, and organelles within the cell (such as lipids, starch, and chloroplasts) [4,5,6,7].

**Figure 1:**
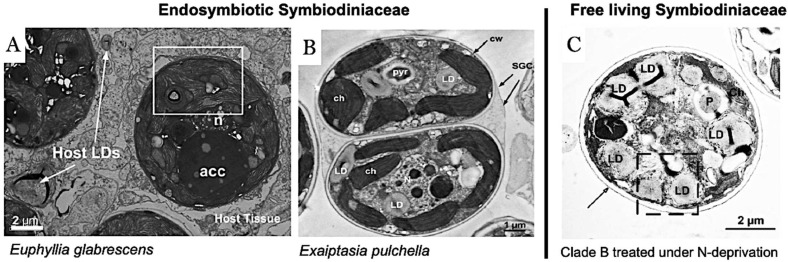
Transmission electron micrographs of endosymbiotic Symbiodiniaceae and free-living Symbiodiniaceae cells. (**A**) Endosymbiotic Symbiodiniaceae reside in the host tissue of *Euphyllia glaberscens*. The white square represented the lipid droplet localization. (**B**) Endosymbiotic Symbiodiniaceae present in the host tissue *Exaipthasia pulchella*. (**C**) Free-living Symbiodiniaceae treated under nitrogen deprivation showed the accumulation of lipid droplets in intracellular cells. Abbreviations: ACC = Accumulation body, LD = Lipid Droplet, Pyr/p = Pyrenoid, Ch = Chloroplast, SGC = Symbiotic Gastrodermal Cell, cw= cell wall, Host LDs = Host Lipid Droplets [Figures relabeled from Pasaribu et al., 2014, Pasaribu et al., 2015, Pasaribu et al., 2016] [9,20,21].

Based on studies of coral-Symbiodiniaceae species relationship over the past decade, lipid droplets have been discovered in these dual organisms [8,9]. It has been reported that host lipid droplets play a key role in endosymbiosis in terms of cellular processes [8]. Compared with lipid droplets in endosymbiotic Symbiodiniaceae species, the lipid droplets remarkably attracted attention due to the reserves energy of endosymbiotic Symbiodiniaceae species to survive under extreme environment stresses [10]. The strategies used in studying coral-Symbiodiniaceae species lipid droplets are distinct compared to other free-living microalgae due to the lipid droplets present in the Symbiodiniaceae species cells within the host [11]. The distribution of lipid droplets in Symbiodiniceae cells is presented in Figure 1. In particular, the involvement of stored energy, such as lipid droplets, in Symbiodiniaceae species has been the primary focus of coral research [10,11]. The accumulation of lipid droplets, produced in intracellular cells of both the coral and the host, has been associated with coral bleaching events. The formation of lipid droplets in Symbiodiniaceae species might correlate with the metabolic instability of the coral host [10]. As a result of the importance of coral survival and health, it is necessary to describe the existence of lipid droplets as a storage energy in endosymbiotic Symbiodiniaceae species.

Lipid droplets (LDs) are organelles that mainly consist of triacylglycerols and sterol esters surrounded by phospholipids and embedded with some proteins [12]. The lipid droplets are also referred to as lipid bodies, oil bodies, or oil globules in other studies [13]. The investigation of lipid droplets has been identified in animals, plants, and microorganisms [14,15,16,17,18]. In plants, seed lipid droplets are exhibited as stable organelles during long-term storage due to integral proteins providing steric hindrance and electronegative repulsion [19]. Environmental stress is one of the factors that disrupt the symbiotic relationship between coral and endosymbiotic Symbiodiniaceae species Changes in extreme environmental conditions such as temperature, ocean acidification, and nutrient levels can trigger the dissociation of the coral-Symbiodiniaceae species symbiosis [9,10]. Exposure to extreme temperatures, both low and high, has been shown to induce physiological changes leading to coral bleaching. A general trend toward extreme temperatures, particularly low and high temperatures, has been observed to elevate lipid droplet levels in Symbiodiniaceae species. On the other hand, nutrients are crucial for maintaining a stable symbiotic relationship in corals and dinoflagellates [10]. The regulation of nutrient sources, including host catabolism and heterotrophy, is transferred from the host to endosymbiotic Symbiodiniaceae species to maintain their development and growth [7,11]. Conversely, coral bleaching can occur under nutrient-limited conditions, impacting the elevation of lipid droplets in endosymbiotic Symbiodiniaceae species [10]. Therefore, when corals are exposed to nitrogen-limited conditions, Symbiodiniaceae species accumulate lipid droplets in their intracellular cells, making coral bleaching more likely. Endosymbiotic Symbiodiniaceae species can survive in nutrient-limited environments, specifically under limited nitrogen levels [10]. The synergistic effects of environmental stressors, including temperature, nutrient availability, and ocean acidification, induce the accumulation of lipid droplets in the symbionts. Additionally, different clades of Symbiodiniaceae species demonstrate varying levels of response in forming lipid droplets in the marine ecosystem. Much less information has been discussed on lipid droplets in endosymbiotic Symbiodiniaceae species.

In this review, we summarize the current knowledge on the biology of lipid droplets in endosymbiotic Symbiodiniaceae species, including their structure, lipid profiles, and associated proteins. Specifically, we further explore the environmental factors that trigger the induction of lipid droplets in response to various environmental cues. Our findings will provide valuable information on the lipid droplet biology of endosymbiotic Symbiodiniaceae species.

## 2. Endosymbiotic Symbiodiniaceae Species Lipid Droplets Structural Components

**Figure 2:**
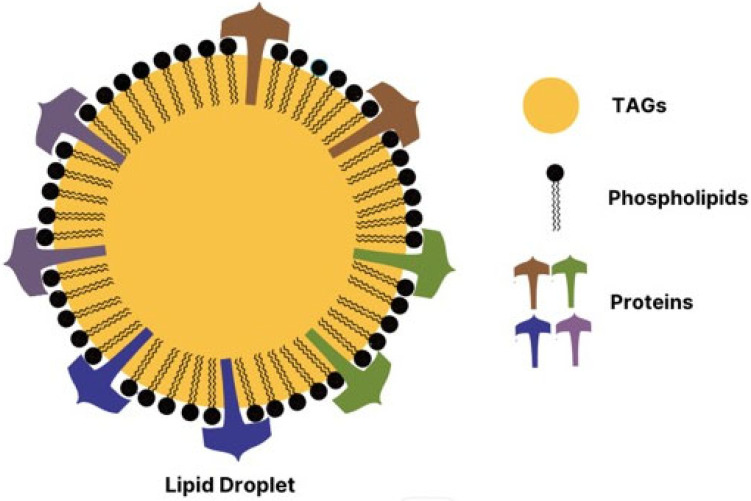
The structure of lipid droplet.

Lipid droplets have been identified in numerous photosynthetic plants and microalgae [22,23]. These Lipid droplets are deposited in seeds, pollen, and non-seeds organs such as leaves, roots, fruits, and stems [24,25,26,27]. Notably, microalgae synthesized the lipid droplets primarily during the stress conditions [28]. Lipid droplets play diverse and crucial roles in biological activities within plants, contributing to response to stress, growth, development, and pathogen resistance [29]. The major constituents of lipid droplets are triacylglycerols (TAGs), surrounded by minor phospholipids and embedded with integral proteins [12,30]. The size of this spherical organelle is 0.5–2.5 micrometers in diameter in diverse species [31,32]. It has been reported that lipid droplet synthesis was initiated at the Endoplasm Reticulum (ER). Briefly, TAGs were synthesized in the smooth ER membrane, and the build-up of TAGs throughout the process formed the spherical structure. The mature spherical lipid droplet is then released from the ER membrane into the cytosol [22]. Figure 2 presents the structure of lipid droplets in plants.

**Figure 3:**
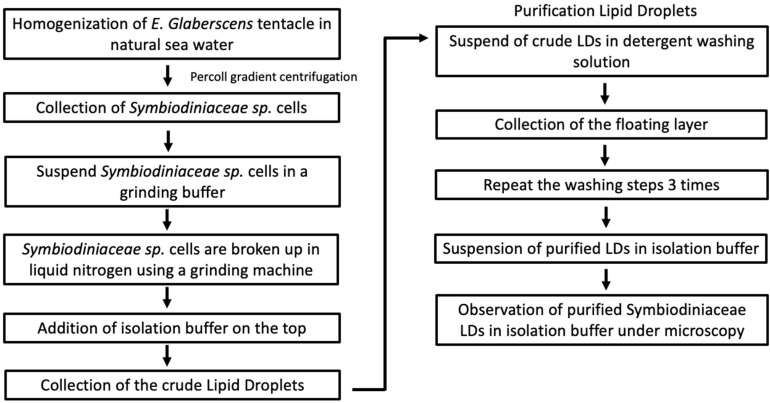
Flow diagram of lipid droplets isolation from endosymbiotic *Symbiodiniaceae* cells.

In plants, the isolated lipid droplets tend to be stable and maintain steric hindrance and electronegative repulsion [33]. Tzen [34] observed the stability of lipid droplets in vivo and in vitro, which never coalesced and aggregated in a cell structure or in the milky top layer during the isolation [34]. The milky top layer of lipid droplets after centrifugation indicated intact lipid droplets of structural integrity and stability, while the lipid droplets were observed under light microscopy [31]. Recently, our knowledge of endosymbiotic Symbiodiniaceae species Lipid droplets have been successfully isolated by centrifugation and sucrose solution process with an average size of 8 micrometers in diameter [33]. Given the key role of lipid droplet isolation in endosymbiotic Symbiodiniaceae species, numerous studies have demonstrated the mechanisms by which the endosymbiotic Symbiodiniaceae species through the validation of the purification process [9,10]. The pure endosymbiotic Symbiodiniaceae species lipid droplets remarkably remained at the top of the layer, and TAGs were the major neutral lipids expressed in the endosymbiotic Symbiodiniaceae species lipid droplets [9]. The stability and electronegative repulsion were maintained as an individual medium when left overnight at 23 °C [33]. The process was an essential step to confirm the presence of lipid droplet protein in the lipid droplets. For instance, in a study, the isolation of lipid droplets through a similar isolation process is analyzed in free-living Symbiodiniaceae species Lipid droplets were successfully isolated from free-living Symbiodiniaceae species with 0.5–2 micrometers in diameter [10]. The proteomic analyses of LDs exhibit the involvement in lipid metabolism, signaling, stress response, and energy metabolism [20,35,36]. It remains to be further investigated by using the analytical and genomics approaches to identify the possible integral protein and genetic functions associated with lipid droplet biogenesis, stress response, and energy metabolism during the symbiotic mutualism association between endosymbiotic Symbiodiniaceae species and coral. The sequential lipid droplet extraction process is presented in Figure 3.

## 3. Neutral Lipid and Fatty Acids Profile of Endosymbiotic Symbiodiniaceae Species Lipid Droplets

The isolation of lipid droplets in endosymbiotic Symbiodiniaceae species was conducted through complex purification techniques. First, the endosymbiotic Symbiodiniaceae species was purified from the tentacle of coral, for example, *Euphylia glabaerscens* [36]. The purification aimed to isolate pure endosymbiotic Symbiodiniaceae species without any contamination from Host cells. The purified endosymbiotic Symbiodiniaceae species underwent the breakage process to extract the LDs from the intracellular cells. The breakage of LDs was purified using Triton x-100 detergent methods for removal of nonspecifically associated proteins from LDs surface [9]. The lipid profile of pure LDs in endosymbiotic Symbiodiniaceae species demonstrated the higher content of TAGs (triacylglycerols) using TLC Chromatography. The conformation of major neutral lipid contents in LDs in endosymbiotic Symbiodiniaceae species was consistent with major LDs observed in land plants, marine plants, and algae [9,27,37,38,39,40]. Furthermore, the isolation of free-living Symbiodiniaceae species of TAG accumulation showed the increasing of lipid bodies demonstrating the composition of lipid droplets of endosymbiotic Symbiodiniaceae species was identified the top fatty acids to be C16:0, C18:0, C22:1, and C22:6. Among them, C22:1 was the most abundant, occupying approximately 30% of total fatty acids. The C12:0, C16:0, C18:0, and C18:1 as the major fatty acids were identified in major lipid Symbiodiniaceae species cells. The proportion of polyunsaturated fatty acids (PUFAs) indicated higher expression in freshly isolated symbiotic cells [9]. This is consistent with a study indicated by Pasaribu et al. 2015 [20], the increasing of lipid droplets in endosymbiotic *Symbiodiniaceae* sp. (clade B). in *Exaiptasia pulchella* demonstrated the increase of de novo synthesis of monounsaturated fatty acids (MUFA) (C16:1, C18:1, C22:1) and polyunsaturated fatty acids (PUFA) (C18:2, C22:6). In contrast, the fatty acid composition of host *Exaiptasia pulchella* cells revealed C12:0, C16:0, and C18:0 as the predominant fatty acids under normal conditions. Notably, following nitrogen deprivation treatment, the host displayed an accumulation of PUFA, with C16:0 still constituting the majority of the fatty acid composition in *E. pulchella* cells [36].

## 4. Major Integral Protein in Endosymbiotic Symbiodiniaceae Species Lipid Droplets of Corals

Over a decade, lipid droplets in Symbiodiniaceae species have been observed that received wide attention from marine biologists [41]. It was reported that the lipid droplets were initially identified through ultramicroscopy, which demonstrated that the lipid droplets are mostly distributed within the cells of endosymbiotic Symbiodiniaceae species [35].

LDs are considered new organelles that are found both in eukaryotic and prokaryotic cells as storage lipids, which consist of neutral lipids. The structures of these lipid compartments are mainly triacylglycerols and sterol esters, which are surrounded by the phospholipid monolayer embedded with some proteins [19]. In plant LDs, the main three classes of lipid droplet protein are identified as Oleosin, Caleosin, and Steroleosin [16,19,27,42]. These proteins consist of the hair-pin structure, which can be anchored into the TAG (triacylglycerols) comprised of a hydrophobic core with a conserved ‘proline knot” and hydrophilic N and C terminal [33]. Therefore, the characterization of the functional lipid droplet protein has the role of preventing their coalescence and controlling the TAG lipolysis [27,42].

Oleosin is a class of alkaline proteins with a relatively low molecular weight of 15–24 kDa that accumulate at the surface of LDs in desiccation-tolerant seeds [43]. The structure of oleosin was described to be comprised of three distinct structural domains: an N-terminal amphipathic domain, a central anti-parallel β-strand domain, and a C-terminal amphipathic α-helical domain [34]. As a hydrophobic protein, oleosin was relatively conserved in most plants, which consists of the motif termed ‘proline knot’ [44]. The function of the proline knot probably played a key role in oleosin structure and targeting LDs. An investigation was carried out to explore the diversity of seed oil bodies, categorizing oleosin oil bodies into High (H) and Low (L) isoforms based on their molecular mass to distinguish among different species. [45,46]. The distinguished isoform of the seed lipid droplet raises curiosity about the function of the High and Low Mr. isoform; even though one of the oleosin proteins can stabilize the lipid droplets in the seed, Pasaribu et al. 2014 [33] reported the G and L-isoform identified in the seed lipid droplets of gymnosperm which both two oleosin H and L isoform were present in the diverse angiosperms. To date, oleosin has a key role in the stability of seed during germination and post-germination [43] and is involved in the lipid biogenesis of LDs.

Caleosin was a known class of protein comprised of the EF-hand calcium-binding protein initially similar to oleosin-associated protein binding in lipid droplets [47]. Caleosin can be found in plants, fungi, and unicellular algae [48,49]. The structure of caleosin was described as an N-terminal hydrophilic domain region with a single Ca^2+^-binding EF hand domain, a central hydrophobic region, and a C-terminal hydrophilic domain [47]. The proline-rich region found in caleosin, which is potentially from the proline knot motif, is unique evidence that the hydrophobic region of the caleosin was shorter than oleosin plays a key role in targeting oil bodies of oleosin [50]. The function of caleosin is involved in signaling and also the oil body assembly or mobilization by the phosphorylation status [51,52]. 

Steroleosin is a minor protein found, firstly, on the sesame seeds, which are comprised of an N-terminal lipid anchoring domain and soluble binding dehydrogenase domain. In terms of steroleosin, it was a unique protein that anchored soluble dehydrogenase located on the surface lipid droplets via its N-terminal segment than oleosin and caleosin were anchored in the central portion of their protein structure [53]. Steroleosin structure was described: an NDPH–binding subdomain, an active site region, and a sterol-binding sub-domain [23]. The Proline Motif knot found in the steroleosin was located in the center of the N-terminal hydrophobic segment. The N-terminal hydrophobic segment was comprised of the two amphipathic α-helicase connected by a hydrophobic segment [54]. The function of sterol eosin was elucidated with the approach of the main comprised of the sterol-binding dehydrogenase, which was known to be involved in signal transduction [55].

Major Lipid Droplet Proteins were studied in several green microalgae, which consist of the major triacylglycerol and phospholipid. Information on the lipid droplets identified from microalgal species is presented in Table 1. The LDs were characterized by identifying the LDs protein duet to elucidate the mechanism and function in green algae. To date, the LDs have been found in algae as major lipid droplet proteins [56,57,58]. Several techniques were applied to induce the LDs in green algae, such as the starch-less mutant, nitrogen starvation, phosphate starvation, light, osmotic stresses, and temperature [59,60,61,62,63,64,65,66,67]. The major lipid droplet protein was found in the green algae with a molecular weight of 17–33 kDa associated with the LDs in green algae [56,57,58,68]. It was reported that the structure of the Major lipid droplet has a similar structure found in the plant oleosin, in which the proline knot motif was anchored into the TAG [56]. The function of the lipid droplet has played a key role in lipid accumulation [56].

Symbiodiniaceae cells (clade C) were successfully isolated from the tentacles of *Euphyllia glabrescens* by a discontinuous personal gradient, and the presence of LDs was confirmed by transmission electron microscopy [33]. Furthermore, stable *Symbiodiniaceae* sp. (clade C). lipid droplets were also successfully purified. It was found that the stability and integrity of the LDs were maintained by electronegative repulsion and steric hindrance, which might be a hint of the presence of surface integral protein. *Symbiodiniaceae* sp. lipid droplet Protein (SLDP) with a molecular weight of 20 kDa was then discovered as the major protein in the purified lipid droplet [36]. Surprisingly, SLDP could be immunologically recognized by anti-cycad caleosin and anti-sesame caleosin antibodies. It was suggested that the stable Symbiodiniaceae species lipid droplets were sheltered by SLDP, which could be a unique structural protein [33]. 

## 5. Environment Factors Elevate Lipid Droplets Accumulation in Endosymbiotic *Symbiodiniaceae*

**Figure 4:**
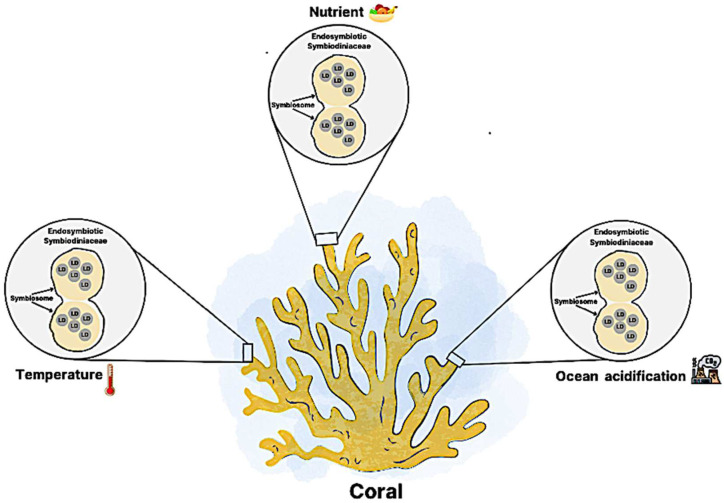
Environment stresses elevate the lipid droplet production in endosymbiotic Symbiodiniaceae species Increased temperature contributes to the lipid droplet accumulation. Nutrient limitation stimulates the formation of lipid droplets in endosymbiotic Symbiodiniaceae species Ocean acidification elevates the lipid droplet accumulation in endosymbiotic Symbiodiniaceae species CO_2_ = Carbon dioxide, LD = Lipid Droplet.

Environmental factors substantially impacted the symbiotic mutualistic association between Symbiodiniaceae species and coral. The rapid changes in the environment have significant implications for the physiology and cellular metabolism of endosymbiotic Symbiodiniaceae species Lipid droplets are known as storage energy that facilitates as one bioindicator in response to different environmental stresses. Several environmental stresses have occurred in the ocean that have impacted symbiotic mutualism, such as nutrient, temperature, and ocean acidification. This review explores current research work on the potential impact of environmental stressors on lipid droplet accumulation in endosymbiotic Symbiodiniaceae species, which is presented in Figure 4.

### 5.1. Nutrient

Nutrient was one of the compulsory points to the mutualism association between *Symbiodiniaceae* and coral. In a decade, debate still ensues regarding the *Symbiodiniaceae* sp. roles reside within the host due to the lack of nutrient relocating established by *Symbiodiniaceae* sp. In 1958, Muscatine and Hand [78] reported that nutrition was transferred by Symbiodiniaceae species to their host. They utilized the ^14^C to label CO_2_ and visualize the movement of the labeled metabolite from algae to host tissue in sea anemone. Even though the revealing of *Symbiodiniaceae* sp. translocated compound to host has been elucidated. The *Symbiodiniaceae* sp. nutrient compound was still a mystery at that time. The revealing of compound content was first elucidated by researchers in identifying the products released by *Symbiodiniaceae* sp., such as Lipid, Glycerol, and Sugar [79,80,81]. In the association between the coral and *Symbiodiniaceae* sp. role of the host as the protective resident is also to provide the nutrients needed by the *Symbiodiniaceae* sp. During endosymbiosis, the host was known to release the nutrients needed by the Symbiodiniaceae species in conducting photosynthesis. In particular, the nutrient was translocated by the host and contained inorganic carbon, inorganic nitrogen, and phosphate [82,83,84]. The nutrient amount translocated by the host to Symbiodiniaceae species raises the speculation that the host controls nutrients released to Symbiodiniaceae species [85,86,87]. Cook & Elia [88] proposed that Symbiodiniaceae species was nutrient-limited by the host, which may regulate the quantity of translocation product in the host. Several pieces of evidence that nutrient deficiency was conducted by the host were studied the increase in the growth rate and density of Symbiodiniaceae species in coral was the nitrogen added to seawater [89,90,91]. Peng et al. [8] demonstrated the assessment of Symbiodiniaceae species metabolic modulation was explored using synchrotron radiation, and nitrogen limitation was observed in the Symbiodiniaceae species residing within the host compared with the free-living Symbiodiniaceae species.

### 5.2. Temperature

Temperature is one of the abiotic factors that directly impact the stability relationship between endosymbiotic Symbiodiniaceae species and its host [92]. The elevated temperature significantly triggered the physiological metabolism and decreased the survival and growth of corals, which often caused bleaching due to the detachment of endosymbiotic Symbiodiniaceae species [93,94]. The morphology of cellular changes such as chloroplast, Golgi complex, and accumulation of lipid droplets was observed after 35 days of experiments in *Cladocopium C3* exposure to heat stress [95]. It was reported that lipid droplets (LDs) elevated in endosymbiotic Symbidiniceae in its host during the coral bleaching impacted the reduction of metabolism exportation to the host by Symbiodiniaceae species [8]. Morphological characteristics of accumulation of lipid droplets (LDs) were observed through scanning electron microscopy that demonstrated the gradual accumulation of lipid droplets distribution while the corals were subjected to partial or severe bleaching conditions [11]. The elevated lipid droplet accumulation was similarly reported in endosymbiotic Symbiodiniaceae Clade B [10]. Lipid droplets were distributed in intercellular Symbiodiniaceae Clade B cells, and numerous inclusion bodies appeared within the lipid droplets exposed to extreme temperature stress at low (15 °C) and high (30 °C) temperatures [20]. During the bleaching process, the surface of lipid droplets was observed to be unstable and modified. It was presumed lipid droplets might be used as energy to survive under extreme temperatures and stress [21,35]. 

### 5.3. Ocean Acidification

The dissolution of CO_2_ in the ocean waters drove the major decrease in ocean pH due to the formation of carbonic acid (HCO_3_^−^) and releasing the Protons (H^+^), so-called Ocean acidification [89,96]. Over a decade, the decrease in ocean waters’ pH reached 0.1 pH unit since the Industrial Revolution, which also impacted the physiological and metabolism of corals [97]. It has been reported that the high concentration of CO_2_ led to coral bleaching by impacting coral survival, growth, and reproduction [98]. Conversely, the physiology of *P. domicornis* larvae in the early stage was not significantly impacted by higher CO_2_ since the usage of energy reserved for larval lipids for survival upon release [99]. As lipids are important for Symbiodiniaceae species under environmental stress, the lipids were stored in lipid droplet formation as reserve energy for survival [21]. Clearly, ocean acidification induced the lipid droplet accumulation in the endosymbiotic Symbiodiniaceae species [100]. Recently, the formation of lipid droplets has also been observed under ultrastructural electron microscopy after 16 days of seawater acidification following the change of fatty acid profile in Symbiodiniaceae species isolated from *Mussismilia braziliensis* [100]. 

## 6. Summary and Future Recommendations

Endosymbiotic Symbiodiniaceae is a coccoid photosynthetic dinoflagellate that forms a symbiotic mutualism relationship that plays a crucial role in providing energy and maintaining host metabolism and health. As the primary producer of energy of its host, the stability of endosymbiotic Symbiodiniaceae species depends on the internal (coral) and external (nutrient, temperature, and ocean acidification) environments. The environmental stresses are the major contributor to the disassociation of endosymbiotic Symbiodiniaceae species led to significant changes in physiology and metabolism. Environmental stress impacts the coral health status associated with the accumulation of lipid droplets in endosymbiotic Symbiodiniaceae species However, the genetic role of lipid droplet-associated proteins in endosymbiotic Symbiodiniaceae species response to stress is still unknown. More advanced studies are required in elucidating the incorporation of various environmental stress conditions to understand the adaptation of coral reefs to climate change and processes that might involve in lipid droplet functional roles in cell metabolism during bleaching events. 

Extreme environmental conditions change the production of lipid droplets in endosymbiotic Symbiodiniaceae species. In fact, one major lipid droplet of endosymbiotic Symbiodiniaceae species (SLDP) has recently been reported from *Euphyllia glabrescens.* It remains unexplored whether the major lipid droplet proteins present in the endosymbiotic Symbiodiniaceae species are from diverse coral species. Due to purifying processes and lesser knowledge of LDs, isolating lipid droplets from endosymbiotic Symbiodiniaceae species is difficult. In the future, the omics approaches could facilitate the exploration of lipid droplets in endosymbiotic Symbiodiniaceae species for further understanding the lipid droplet biogenesis and identifying major lipid droplet proteins in endosymbiotic Symbiodiniaceae species These studies may provide novel opportunities for the mobilization of neutral lipids encapsulated in endosymbiotic Symbiodiniaceae species lipid droplets by LD-anchored proteins.

## Figures and Tables

**Table 1:** Summary of Lipid droplet proteins identified from microalgal species.

Microalgae Group	Species	Lipid Droplet (LD) Proteins	References
Streptophyta	*Spirogyra grevilleana*	Oleosin	[69]
Chlorophyta	*Cosmarium turpinii*		
Chlorophyta	*Closterium acerosum*		
Chlorophyta	*Auxenochlorella protothecoides*	Caleosin	[36,49]
Chlorophyta	*Chlorella vulgaris*		[70]
Chlorophyta	*Chlamydomonas reinhardtii*	Major Lipid Droplet Protein (MLDP)	[56,71]
Chlorophyta	*Dunaiella salina*		[58]
Chlorophyta	*Scenedesmus quadricauda*		[72]
Chlorophyta	*Chromochloris zofingiensis*		[40]
Chlorophyta	*Lobosphaera incisa*		[73]
Chlorophyta	*Haematococcus pluvialis*	Haematococcus oil globule protein (HOGP)	[57]
Chlorophyta	*Nannochloropsis oceanica*	Lipid Droplet Surface Protein (LDSP)	[74,75]
Diatom	*Phaeodactylum tricornutum*	LD-associated protein (PtLDP1)	[76]
	*Fistulifera solaris*	A homolog of oleosome-associated-protein 1 (DOAP1)	[77]
Dinoflagellate	*Symbiodiniceae* Clade C from *Euphyllia glabrescens*	Symbiodinium lipid droplet protein (SLDP)	[9]

## Data Availability

This study did not report any data.
